# LZ-101, a novel derivative of danofloxacin, induces mitochondrial apoptosis by stabilizing FOXO3a via blocking autophagy flux in NSCLC cells

**DOI:** 10.1038/s41419-019-1714-y

**Published:** 2019-06-19

**Authors:** Yongjian Guo, Yue Zhao, Yuxin Zhou, Xiaoqing Tang, Zhiyu Li, Xiaotang Wang

**Affiliations:** 1 Department of Chemistry and Biochemistry, Florida International University, Miami, FL USA; 2 State Key Laboratory of Natural Medicines, Jiangsu Key Laboratory of Carcinogenesis and Intervention, China Pharmaceutical University, 24 Tongjiaxiang, 210009 Nanjing, The People’s Republic of China; 3 State Key Laboratory of Natural Medicines, Department of Natural Medicinal Chemistry, China Pharmaceutical University, 24 Tongjiaxiang, 210009 Nanjing, The People’s Republic of China

**Keywords:** Drug development, Macroautophagy

## Abstract

Non-small-cell lung carcinoma (NSCLC) continues to be a vital disease worldwide for its high incidence and consequent mortality rate. In this study, we investigated the anti-cancer effect of LZ-101, a new derivative of danofloxacin, against non-small-cell lung cancer and the underlying mechanisms. In vitro, LZ-101 inhibited the viability of human non-small cell lung cancer cell lines. We demonstrated that LZ-101 induced mitochondrial-mediated apoptosis by increasing Bax/Bcl-2 ratio, loss of mitochondrial membrane potential (*ΔΨm*), release of cytochrome *c* (Cyt *c*) and apoptosis-inducing factor (AIF) in A549 cells. Further research illuminated that LZ-101 induced apoptosis was related to the activation of FOXO3a/Bim pathway. Moreover, we found that LZ-101 increased the stability of FOXO3a by blocking autophagy-dependent FOXO3a degradation. However, inhibition of autophagosome formation abolished FOXO3a stabilization and apoptosis induced by LZ-101. In vivo, LZ-101 exerted a remarkable anti-tumor activity with high safety in xenograft model inoculated A549 tumor through the same mechanism as in our in vitro study. In conclusion, our findings indicated that LZ-101 induces mitochondrial apoptosis and stabilizes FOXO3a by blocking autophagy flux.

## Introduction

Lung cancer is a leading cause of cancer-related mortality in a global perspective, of which non-small-cell lung carcinoma (NSCLC) has the highest risk of causing lung cancer-related deaths. Almost 80% of lung cancer-related deaths occur in patients with non-small-cell lung cancer^1,2^. Despite recent advances in therapeutic regimen, the curative effect is not so inspiring because of the poor outcome^3^. Consequently, it is indispensable to develop more effective therapies against lung cancer.

There are two major apoptosis pathways, the extrinsic and intrinsic (mitochondria-associated) pathways, in cytoplasm. Intrinsic apoptosis could be regulated via mitochondrial pathway characterized by the release of cytochrome *c* (Cyt *c*) and activation of caspase^4^. The Bcl-2 family proteins, anti-apoptotic proteins (such as Bcl-2 and Bcl-xL), pro-apoptotic proteins (such as Bax and Bak), and Bcl-2 homology 3-only (BH3-only) proteins (such as Bim, Bad, and Puma), play a crucial role in intrinsic apoptotic process. The apoptotic stimuli activate specific BH3-only proteins to engage anti-apoptotic proteins and release downstream pro-apoptotic proteins, leading to increased mitochondrial outer membrane permeability and eventually cell death induced by triggering caspase cascade^5^. Subsequently, cytochrome *c* is released from mitochondria into cytoplasm which can trigger the caspase cascade, leading to cell death. Bim acts as direct activators of Bax and Bak by interacting with them. Bim is also involved in p53-dependent and p53-independent apoptosis^6^. Recent studies have implicated that Bim may serve as a potential target for anti-cancer chemotherapeutics since it plays an important role in apoptosis^7,8^.

The FOXO subfamily of transcription factors is evolutionarily conserved, including FOXO1, FOXO3a, FOXO4, and FOXO6^9^. FOXO proteins can regulate multiple target genes involved in tumor suppression, such as Bim, FasL, p27kip1, cyclin D and GADD45^10–13^. FOXO3a, the most important transcription factor in FOXO family, was phosphorylated by Akt at Thr32, Ser253, and Ser315, leading to FOXO3a translocate from nucleus to cytoplasm and is consequently degraded by proteasome^14^. The proteasome inhibitor MG132 increases the stability of FOXO3a and induces apoptosis in thyroid cancer cell^15^. In addition, studies have reported that FOXO3a is a substrate for autophagy^16^. This suggests that FOXO3a degradation depends not only on the proteasome pathway, but also on autophagy activation.

LZ-101 is a derivative of danofloxacin that has been developed specifically for veterinary use^17^. Danofloxacin has been widely used for the treatment for respiratory disease and urinary tract infections in animals, such as chicken and buffalo^18,19^. However, studies have shown that danofloxacin induces apoptosis by inducing oxidative stress in renal tubular cells, epithelial cell line (LLC-PK1). This study showed that danofloxacin exhibited apoptosis-inducing effects. While the effect of danofloxacin derivative LZ-101 on apoptosis is still unclear. This study demonstrated that LZ-101 induced apoptosis in A549 human non-small-cell lung cancer cells and inhibited tumor growth with low systemic toxicity in BALB/c mice bearing A549 tumor through mitochondria-associated pathway by stabilizing FOXO3a via blocking autophagy flux. Our results showed that LZ-101 exhibits remarkable anti-tumor activity and is promising to serve as an effective candidate for the treatment of human non-small-cell lung cancer.

## Results

### LZ-101 inhibited cell viability in human non-small-cell lung cancer cells

**Fig. 1:**
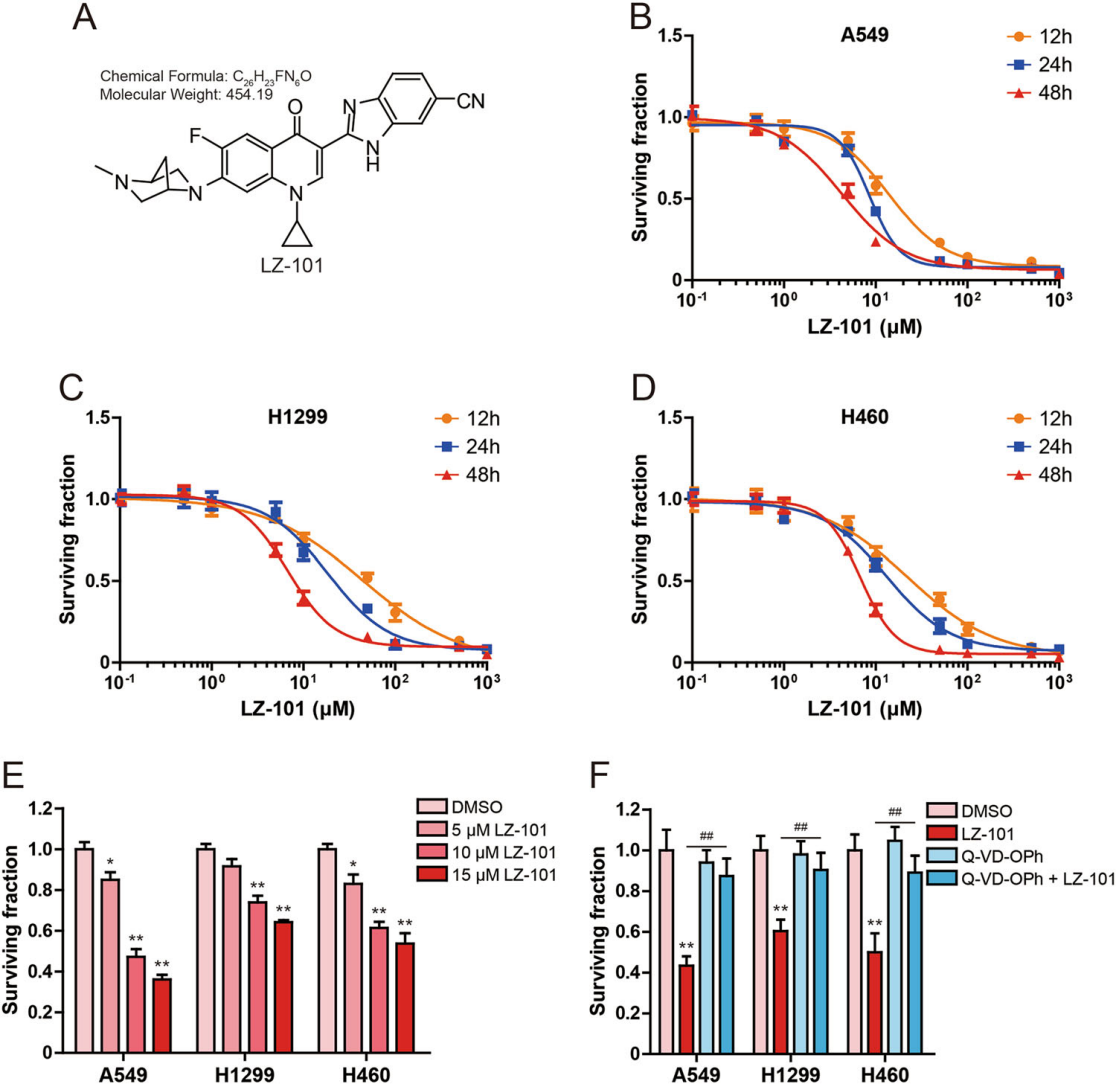
LZ-101 inhibits the viability of human non-small-cell lung cancer cells. **a** LZ-101 molecular structure (C_26_H_23_FN_6_O, Molecular Weight: 454.19). Effect of LZ-101 on the viability of human non-small cell lung cancer cells. MTT assay was used to detect cell viability after treatment of different concentrations of LZ-101 for 12 h, 24 h, and 48 h in A549 (**b**), H1299 (**c**) and H460 (**d**). **e** Cell viability was detected after treatment of 5, 10, and 15 μM LZ-101 for 24 h in A549, H1299, and H460 cells. **f** Cell viability was detected after treatment of 20 μM Q-VD-OPh or 15 μM LZ-101 for 24 h in A549, H1299, and H460 cells. Data are presented as mean ± SD. ^*^P < 0.05, ^**^P < 0.01 compared with DMSO group. ^##^*P* < 0.01

The chemical structure of LZ-101 was shown in Fig. 1a. To evaluate the inhibitory effect of LZ-101 on human non-small-cell lung cancer cells including A549, H1299, and H460 cells, we investigated its effect on cell viability at different concentrations with varying lengths (12, 24, or 48 h) of treatment. The IC_50_ (the concentration of drug inhibiting 50% of cells) values for A549 cells were 13.95 ± 2.24, 8.61 ± 0.75, and 4.28 ± 0.42 μM, respectively, after 12, 24, and 48 h treatment (Fig. 1b). Whereas, the IC_50_ values for H1299 were 44.47 ± 6.54, 18.47 ± 0.86, and 6.75 ± 0.58 μM, respectively, after 12, 24, and 48 h treatment (Fig. 1c). In H460 cells, the IC_50_ values were 22.49 ± 4.52, 13.15 ± 1.02, and 6.80 ± 0.72 μM, respectively, after 12, 24, and 48 h treatment (Fig. 1d). As shown in Fig. 1e, treatment with 5, 10, and 15 μM LZ-101 for 24 h significantly inhibited the surviving of A549, H1299, and H460 cells with A549 cells being the most sensitive to LZ101. Therefore, A549 cell line was chosen for further experiments with 5, 10, and 15 μM of LZ-101 treatment for 24 h. To explore the mechanism of LZ-101 inhibiting A549, H1299, and H460 cells survival, cells were also treated with a pan-caspase inhibitor, Q-VD-OPh, during LZ-101 treatment. Survival inhibition of LZ-101 was significantly inhibited in A549, H1299, and H460 cells, when caspase activity was inhibited by Q-VD-OPh (Fig. 1f). This suggests that LZ-101 inhibited the survival of human non-small-cell lung cancer cells by triggering apoptosis.

### LZ-101 induced mitochondrial apoptosis in A549 cells

**Fig. 2:**
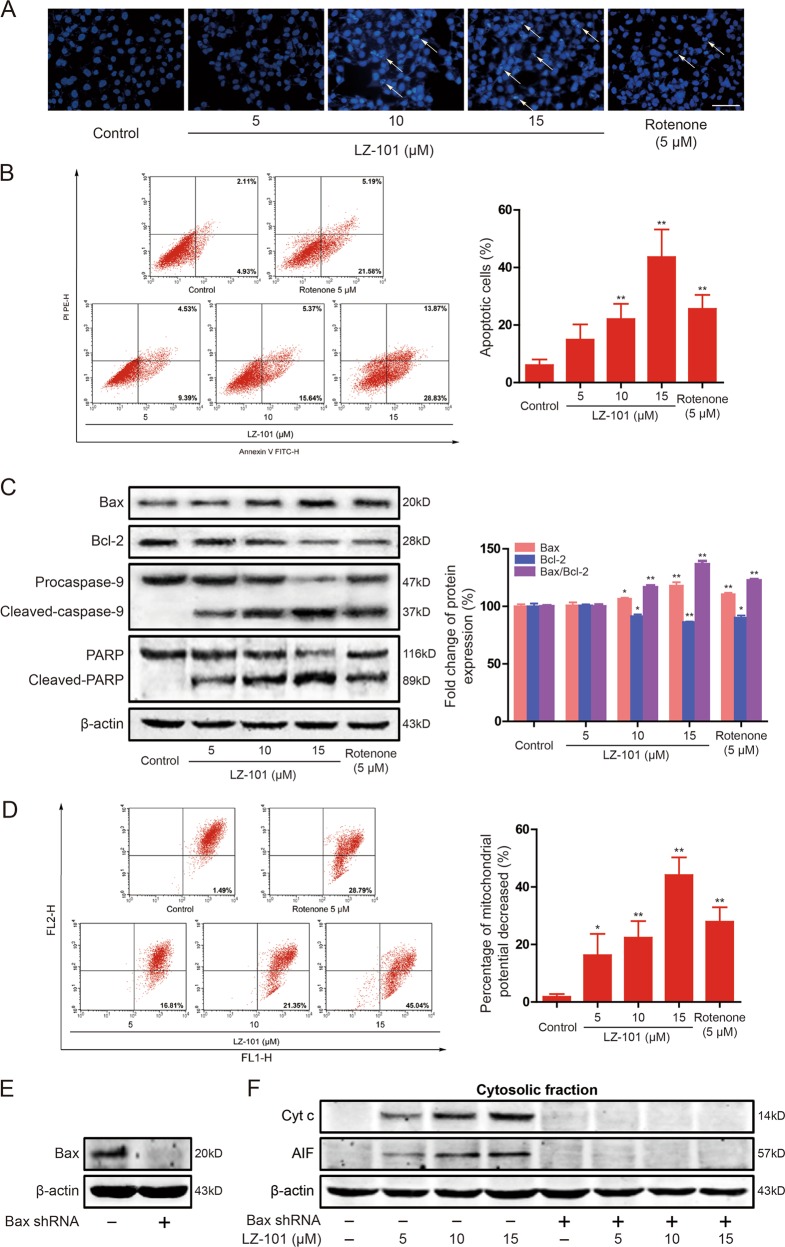
LZ-101 induces mitochondrial apoptosis in A549 cells. A549 cells were exposed to LZ-101 (5, 10, and 15 μM) or Rotenone (5 μM) for 24 h. **a** DAPI staining was used to detect the apoptosis. Scale bars, 50 µm. **b** Induction of apoptosis were measured by Annexin-V/PI double-staining assay (left). Histograms shows the distribution of apoptotic cells for three independent experiments (mean ± SD) (right). **c** The levels of Bax, Bcl-2, caspase-9, and PARP were assessed by western blot. Ratio of Bax/Bcl-2 expression using densitometric analysis. **d** The change of *ΔΨ*_*m*_ as detected by flow cytometry using JC-1 staining. **e** Bax were detected by western blot after transfection of Bax shRNA into A549 cells. **f** The release of Cyt *c* and AIF from mitochondria into cytoplasm was measured by Western blot assay. Data are presented as mean ± SD. ^*^P < 0.05, ^**^P < 0.01 compared with DMSO group

To detect the apoptosis induced by LZ-101 in A549 cells, DAPI staining assay and Annexin-V/PI staining were used with rotenone as positive control. In DAPI staining assay, control cells emitted blue fluorescence with consistent nucleus intensity and showed typical homogeneous distribution of chromatin in nucleus. After LZ-101 treatment, cells presented morphological features of bright apoptotic bodies and nuclear condensation (Fig. 2a arrows). These features appeared more frequently with increasing concentrations of LZ-101. To further confirm the apoptosis induced by LZ-101, Annexin-V/PI staining assay was used. As shown in Fig. 2b, increased apoptotic was detected after LZ-101 treatment for 24 h. The percentage of apoptotic cells increased from 6.07% ± 1.62% to 14.92% ± 4.32%, 22.07% ± 4.32%, and 43.62% ± 7.83%, respectively. Furthermore, the apoptosis related proteins such as Bcl-2, Bax, caspase-9 and PARP were investigated by Western blots. After treatment with LZ-101 for 24 h, the apoptotic protein Bax expression increased while the anti-apoptotic protein Bcl-2 expression decreased in a concentration-dependent manner (Fig. 2c). The ratio of Bax/Bcl-2 is crucial for the activation of the mitochondrial apoptotic pathway. The ratio of Bax/Bcl-2 was increased by different concentrations of LZ-101 treatment. Besides, Caspase-9 and PARP cleavage were activated significantly after LZ-101 treatment (Fig. 2c). These data indicated that LZ-101 induced apoptosis in A549 cells.

In order to elucidate the mechanism of LZ-101-induced apoptosis in A549 cells, we investigated the effect of LZ-101 on mitochondrial function. Changes in mitochondrial functions are essential for mitochondrial apoptotic pathway. In early apoptosis stage, the change of mitochondrial membrane potential (*ΔΨm*) is a marker for mitochondrial dysfunction. Accordingly, we used a fluorogenic probe JC-1 staining cells to detect the change of *ΔΨm* after LZ-101 treatment. Flow cytometric analysis revealed that A549 cells became more susceptible to mitochondrial membrane depolarization after LZ-101 treatment (Fig. 2d). We observed almost 50% decrease in *ΔΨm* with 15 μM LZ-101, compared with the control group. Multidomain pro-apoptotic proteins like Bax and Bak play pivotal roles in the release of apoptogenic proteins, such as cytochrome *c* and AIF, from the mitochondria into the cytosol in response to apoptotic stimuli^20^. To study the involvement of Bax in LZ-101-induced apoptosis, we used Bax shRNA to diminish the expression of Bax (Fig. 2e). As shown in Fig. 2f, cytochrome *c* and AIF were released from mitochondria upon LZ-101 treatment whereas their release was almost completely blocked in Bax knockdown cells and β-actin levels of all the fractions were similar. Taken together, our findings suggested that LZ-101 induced apoptosis was mediated by mitochondrial dysfunction in A549 cells.

### The up-regulation of FOXO3a expression was involved in LZ-101 induced apoptosis of mitochondrial

**Fig. 3:**
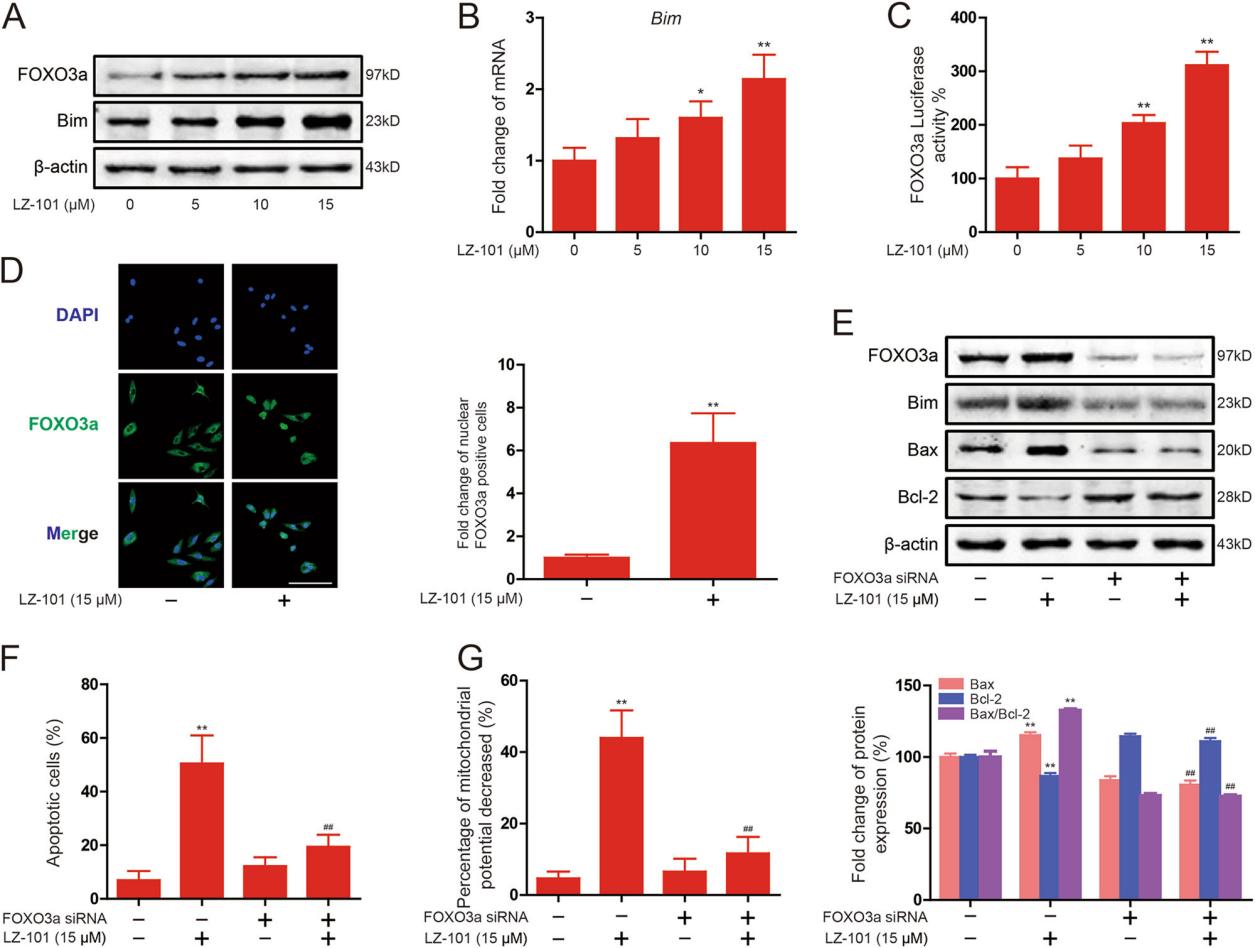
LZ-101 induces mitochondrial apoptosis by up-regulating FOXO3a expression in A549 cells. **a** The levels of FOXO3a and Bim were assessed by Western blot after 5, 10, and 15 μM LZ-101 treatment for 24 h. **b** Bim mRNA was measured by real-time PCR following treatment with 5, 10, and 15 μM LZ-101 for 24 h in A549 cells. β-actin was used as internal control. The relative levels were calculated to β-actin mRNA expression. **c** The transcriptional activities of FOXO3a in A549 cells co-transfected with pGMFOXO-Lu and pRL-TK Renilla with LZ-101. Luciferase activity was determined 24 h post-treatment by promega dual luciferase reporter assay system, normalized against values for the corresponding pRL-TK Renilla activity. **d** Immunofluorescence staining of FOXO3a in A549 cells (treated with 15 μM LZ-101) was carried out to test the effect of LZ-101 on FOXO3a protein nuclear translocation. Scale bars, 50 µm (left). Histograms shows the fold change of nuclear FOXO3a positive cells for three independent experiments (mean ± SD) (right). FOXO3a siRNA was transfected into A549 cells. The cells were cultured in serum free medium overnight. After 8 h, cells were treated with 15 μM LZ-101 for 24 h. **e** FOXO3a, Bim, Bax, and Bcl-2 were detected by Western blot (top). Ratio of Bax/Bcl-2 expression using densitometric analysis (bottom). **f** Annexin-V/PI staining assay and **g** the change of *ΔΨ*_*m*_ were measured by flow cytometry. Data are presented as mean ± SD. ^*^P < 0.05, ^**^P < 0.01 compared with DMSO group; ^*#*^*P* < 0.05, ^*##*^*P* < 0.01 compared with LZ-101 treatment group

When cells receive intrinsic apoptosis signals, the pro-apoptotic BH3-only proteins, such as Bim, Bad, Noxa, and Puma, will be activated. These proteins not only inhibit the activity of anti-apoptotic protein (Bcl-2, Bcl-xL, and Mcl-1), but also activate the pro-apoptotic protein (Bax and Bak), which form an oligomer and subsequently punch pores in the outer mitochondrial membrane, eventually, leading to permeabilization of mitochondrial outer membrane^6^. As mentioned before, although pro-apoptotic BH3-only protein Bim plays a crucial role in apoptosis, it could be regulated by FOXO3a at transcriptional level. Therefore, we investigated the effect of LZ-101 on this signaling pathway. Western blots analysis showed that Bim increased in A549 cells after LZ-101 treatment for 24 h (Fig. 3a). This result confirms that FOXO3a regulated the transcription of Bim. Moreover, we found that LZ-101 up-regulated FOXO3a protein expression in A549 cells (Fig. 3a). The change of mRNA level of Bim, tested by Real-time PCR, was consistent with that of the protein levels (Fig. 3b). Furthermore, Luciferase reporter gene assay suggested that LZ-101 increased transcriptional activity of FOXO3a in a concentration-dependent manner (Fig. 3c). LZ-101 also promoted FOXO3a to translocate into the nucleus (Fig. 3d). Therefore, LZ-101 up-regulates the expression of FOXO3a and promotes the transcription of Bim.

To further explore the mechanism of LZ-101-induced apoptosis and determine the roles of FOXO3a activation in this process, we detected the influence of LZ-101 on Bax and Bcl-2 expression after FOXO3a siRNA transfection. Western blot results showed that the protein level of Bax and Bcl-2 were notably regulated by LZ-101. However, these effects of LZ-101 were obviously reversed by the knockdown of FOXO3a (Fig. 3e). Remarkably, the ratio of Bax/Bcl-2 increased by LZ-101 was also reversed. Moreover, Flow cytometry analysis demonstrated that the percentage of apoptotic cells in LZ-101 group transfected with FOXO3a siRNA reduced from 50.47% ± 8.59% to 19.44% ± 3.63%, compared with LZ-101 group without transfection with FOXO3a siRNA (Fig. 3f). The decrease of mitochondrial potential was inhibited in LZ-101 group transfected with FOXO3a siRNA compared with LZ-101 group (Fig. 3g). In conclusion, the up-regulation of FOXO3a expression was involved in the induction of mitochondrial apoptosis by LZ-101.

### LZ-101 increased the stability of FOXO3a in a proteasome-independent manner

**Fig. 4:**
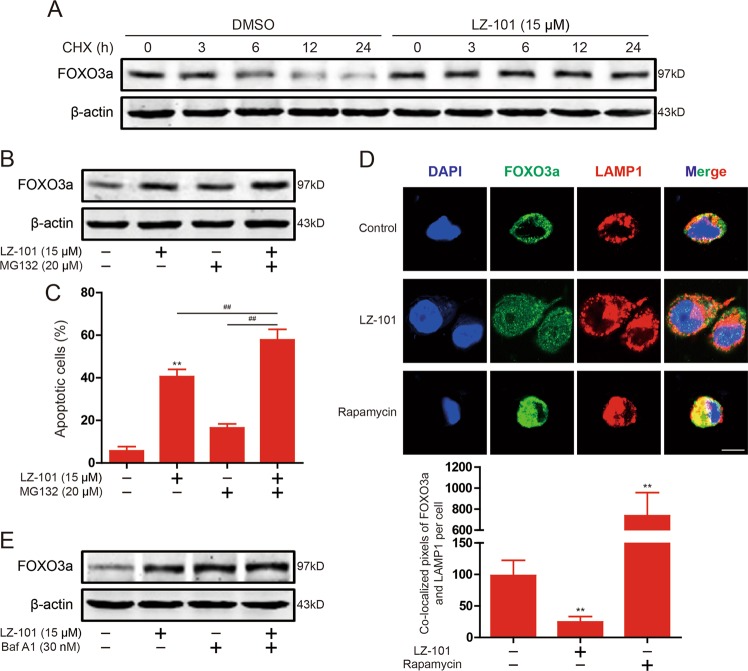
LZ-101 stabilized FOXO3a proteins in a proteasome-independent manner in A549 cells. **a** Degradation dynamics of FOXO3a following a time course CHX treatment in A549 cells treated with 15 μM LZ-101. **b** FOXO3a proteins were stabilized in the presence of MG132 (20 μM) in A549 cells treated with 15 μM LZ-101. **c** Annexin-V/PI staining assay measured by flow cytometry in the presence of MG132 (20 μM) in A549 cells treated with 15 μM LZ-101. **d** Confocal microscopy of A549 cells treated with 15 μM LZ-101 or 100 nM rapamycin, immunostained for FOXO3a (green) and LAMP-1 (red). Scale bars, 10 µm (top). Histograms shows the co-localized pixels of FOXO3a and LAMP1 per cell for three independent experiments (mean ± SD) (bottom). **e** FOXO3a was detected by Western blot after 15 μM LZ-101 and 30 nM bafilomycin A1 treatment. Data are presented as mean ± SD. ^*^P < 0.05, ^**^P < 0.01 compared with DMSO group; ^*#*^*P* < 0.05, ^*##*^*P* < 0.01

To further investigate the molecular mechanism by which LZ-101 up-regulates FOXO3a expression, de novo protein synthesis inhibitor cycloheximide (CHX) and proteasome inhibitor MG132 were used, respectively. After treatment of CHX, FOXO3a was more stable in A549 cells treated by LZ-101 compared with A549 cells treated by DMSO (Fig. 4a), indicating that LZ-101 interfered with FOXO3a degradation. In support of this observation, when MG132 (20 μM) was added to A549 cells, increased FOXO3a levels were observed, whereas LZ-101 unexpectedly further increased FOXO3a levels in A549 cells treated with MG132 (Fig. 4b). Further detection of apoptosis levels showed that LZ-101 significantly increased apoptosis induced by MG132 (Fig. 4c). These results suggested that LZ-101 interfered with FOXO3a degradation in a proteasome-independent manner. In addition to proteasome degradation of proteins in cells, the autophagic lysosomal pathway may also be involved in protein degradation. To confirm this hypothesis we detected the co-localization of FOXO3a and LAMP1 in A549 cells. As shown in Fig. 4d, LZ-101 indeed inhibited the co-localization of FOXO3a and LAMP1. To further confirm FOXO3a is degraded by the autophagic lysosomal pathway, an autophagolysosome inhibitor, bafilomycin A1, was used. After treatment with bafilomycin A1, FOXO3a degradation was inhibited, combination of bafilomycin A1 with LZ-101 could not further inhibit FOXO3a degradation (Fig. 4e). These results suggested that LZ-101 increased the stability of FOXO3a through the autophagic lysosome pathway rather than the ubiquitin proteasome pathway.

### LZ-101 blocked autophagy flux in A549 cells

**Fig. 5:**
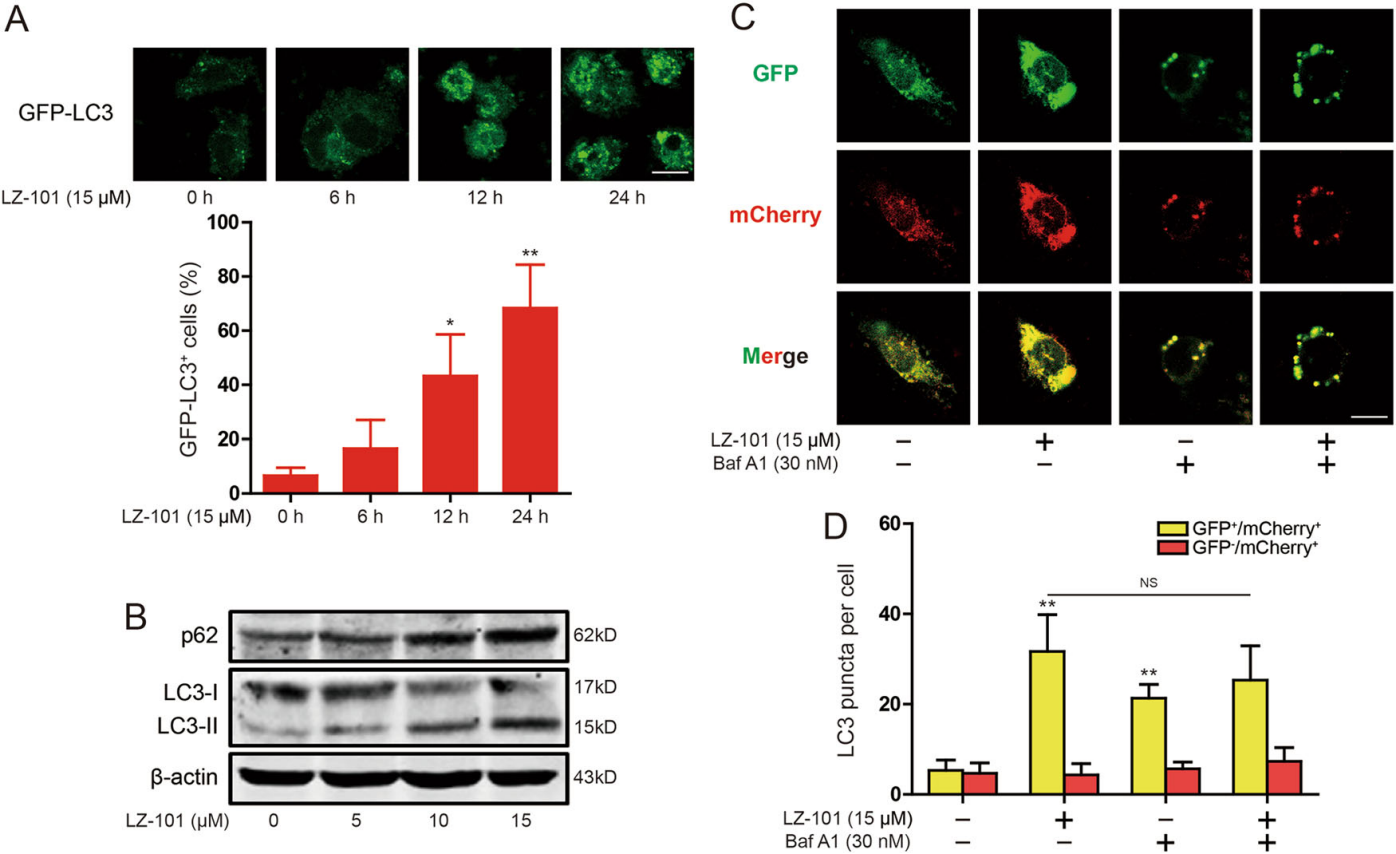
LZ-101 blocked autophagy flux in A549 cells. **a** Induction of GFP^+^ dots in A549 cells expressing GFP-LC3 treated with 15 μM LZ-101. Images of individual cells and quantified of GFP-LC3^+^ cells. Scale bars, 10 µm. **b** p62 and LC3 were detected by western blot in A549 cells treated with 15 μM LZ-101. **c** A549 cells expressing mCherry-GFP-LC3 were observed under a confocal microscopy after 15 μM LZ-101 and 30 nM bafilomycin A1 treatment. Representative images are shown. Scale bars, 10 µm. **d** The average numbers of yellow or red puncta were obtained from three countings. Data are presented as mean ± SD. ^*^P < 0.05, ^**^P < 0.01 compared with DMSO group

In Fig. 4d, 100 nM rapamycin remarkably increased the co-localization of FOXO3a and LAMP1 in A549 cellls. This is consistent with the study that FOXO3a is an autophagy substrate^16^. To further investigate the molecular mechanism by which LZ-101 increased the stability of FOXO3a, we investigated the effect of LZ-101 on autophagy. LC3-I and LC3-II are both produced post-translationally, but LC3-I is cytosolic and LC3-II, the hallmarks of autophagy, is membrane bound and forms the autophagosome^21^. Treatment with 15 μM LZ-101 increased GFP-LC3 puncta accumulation (Fig. 5a). Then, we assessed LC3 in the cell lysates by immunoblot analysis. We found that LC-II protein levels gradually increased following treatment with LZ-101 in A549 cells (Fig. 5b), indicateing that LZ-101 increased autophagosome formation in A549 cells. Since enhanced autophagosome accumulation could be caused either by promoted autophagosome formation or by suppressed autophagosome degradation, we determined whether a complete autophagic flux occurred after LZ-101 treatment. As shown in Fig. 4b, the degradation of p62 was significantly suppressed in A549 cells with LZ-101 treatment. To further assess the status of autophagic flux, we used the mCherry-GFP-LC3 construct. As the more stable of mCherry in acidic conditions compared with GFP, autophagic flux can be determined by the appearance of more GFP^-^ mCherry^+^ (red) puncta. After treatment with LZ-101, large-sized GFP^+^ mCherry^+^ (yellow) puncta were observed, while the yellow puncta did not change significantly after bafilomycin A1 treatment (Fig. 5c, d). These results indicated that LZ-101 blocked autophagy flux in A549 cells by inhibiting autophagosome degradation.

### Inhibition of autophagosome formation abolished the induction of apoptosis by LZ-101

**Fig. 6:**
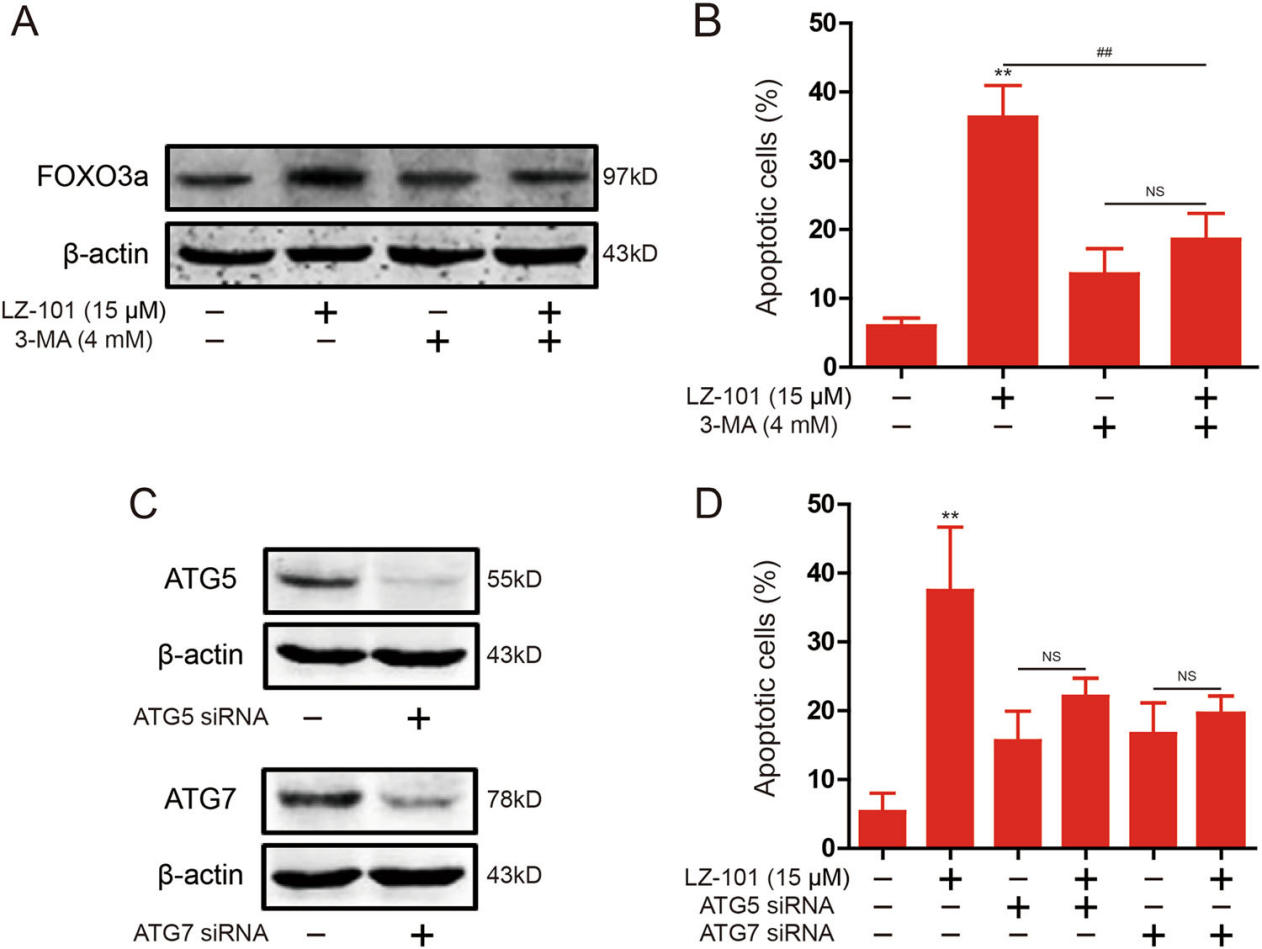
Apoptosis induced by LZ-101 was abolished after inhibition of autophagosome formation in A549 cells. **a** FOXO3a was detected by western blot in the presence of 3-MA (4 mM) in A549 cells treated 15 μM LZ-101. **b** Annexin-V/PI staining assay by flow cytometry in the presence of 3-MA (4 mM) in A549 cells treated with 15 μM LZ-101. **c** ATG5 and ATG7 were detected by western blot after transfection of ATG5 or ATG7 siRNA into A549 cells. **d** Annexin-V/PI staining assay by flow cytometry in A549 cells treated with 15 μM LZ-101 and transfected ATG5 or ATG7 siRNA. Data are presented as mean ± SD. ^*^P < 0.05, ^**^P < 0.01 compared with DMSO group

To investigate the role of autophagy in the induction of apoptosis by LZ-101, we blocked the formation of autophagosomes with 3-MA. After treatment with 3-MA, the effect of LZ-101 to stabilize FOXO3a abolished (Fig. 6a). In addition, the effect of LZ-101 induced apoptosis was similarly abolished after treatment with 3-MA (Fig. 6b). In the process of autophagy, ATG5 and ATG7 are required for the formation of the autophagosome^22^. As shown in Fig. 6c, we silenced the expression of ATG5 or ATG7 through transfecting siRNA. The induction of apoptosis was abolished after diminishing the expression of ATG5 or ATG7 in LZ-101-treated A549 cells (Fig. 6d). Thus, blocking the formation of autophagosome abolished LZ-101 induced apoptosis of A549 cells.

### LZ-101 exerted anti-tumor effect with low toxicity in A549 inoculated xenograft mice

**Fig. 7:**
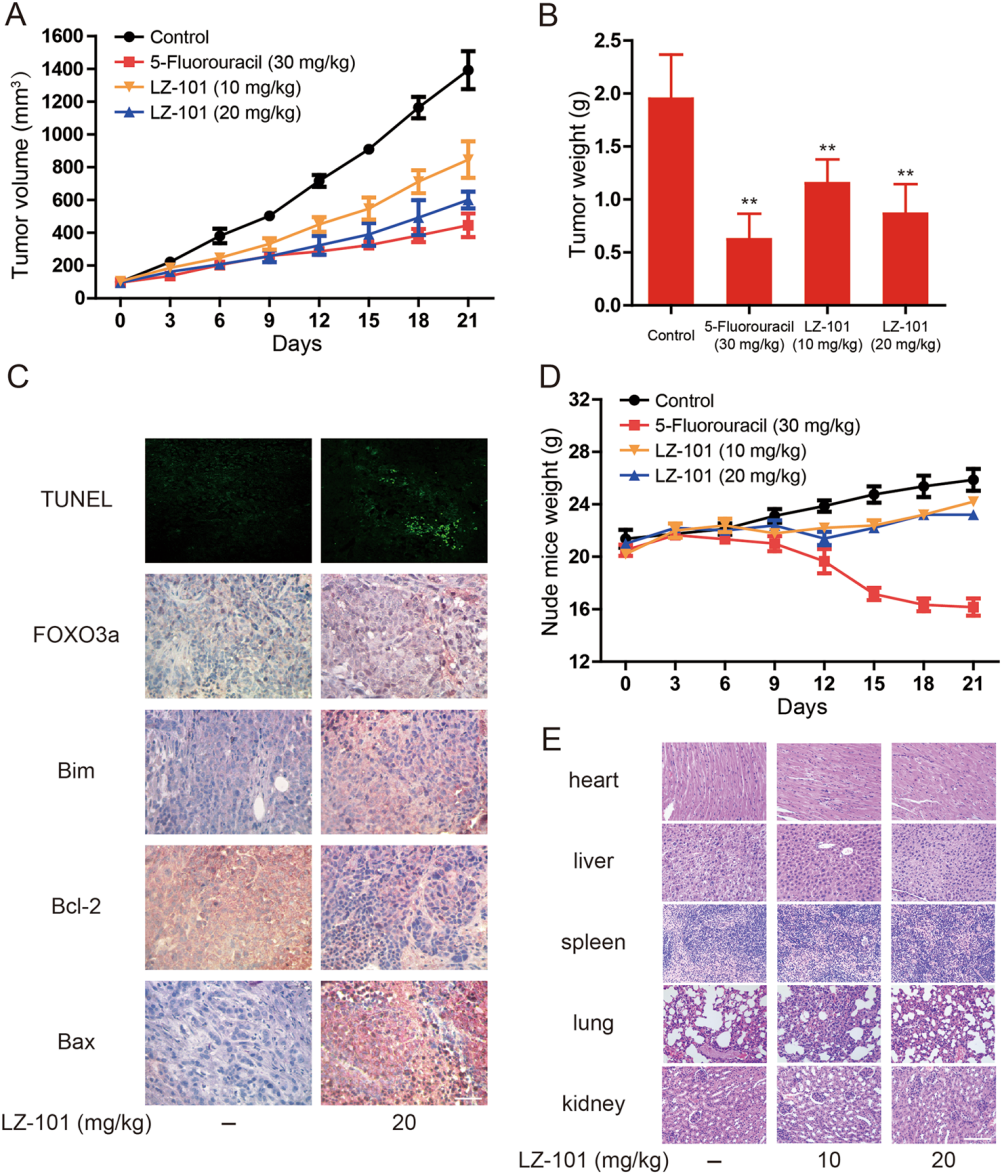
LZ-101 has a potential anti-tumor effect and low toxicity in vivo. **a** Tumor volumes of control, 5-fluorouracil, LZ-101 treatment groups were measured and calculated every 3 days. **b** Weight of tumor in control, 5-fluorouracil, LZ-101 treatment groups. ^*^P < 0.05, ^**^P < 0.01 compared with control group. **c** DNA damage and FOXO3a, Bim, Bcl-2, Bax expression detected by TUNEL assay and immunohistochemistry in tumor xenograft tissues. Scale bars, 50 µm. **d** Nude mice weight was recorded every 3 days. **e** H&E stained main organs of mice from treated and control sets to evaluate the toxicity of LZ-101. Scale bars, 50 µm

The xenograft mice transplanted with A549 cells were used to evaluate the anti-tumor effect of LZ-101 in vivo. The tumor volume of mice with LZ-101 treatment was smaller than that of mice in control group but larger than that of mice in 5-fluorouracil-treated group at the same measurement day (Fig. 7a). Moreover, the tumor weight of LZ-101-treated mice was significantly smaller than that of the control group (Fig. 7b). Accordingly, the inhibitory rates of 5-fluorouracil, 10 mg/kg and 20 mg/kg LZ-101 groups were 68.05%, 40.86%, and 55.62%, respectively (Table 1). The TUNEL assay was performed to detect apoptotic cells in tumor tissues. The results indicated that DNA damage in tumor tissues was induced by LZ-101. In addition, immunohistochemical analysis showed that Bcl-2 expression were decreased while FOXO3a, Bim and Bax expression were increased after LZ-101 treatment (Fig. 7c). Furthermore, we monitored the toxicity of LZ-101 during the entire in vivo experiment. LZ-101 treatment (21 days) did not cause any physical abnormalities. Hematological parameters showed that there were no notable changes in the analyzed parameters in the experimental animals, as listed in Table 2 with the standard ranges for mice. Besides, there was no significant difference in the average body weight of LZ-101-treated mice compared with that of the control mice (Fig. 7d). H&E staining (Fig. 7e) showed no morphological changes in the organs of mice with LZ-101 treatment. Taken together, our results suggested that LZ-101 inhibited tumor growth via FOXO3a activation in xenograft mice bearing A549 tumor with low toxicity in vivo.

**Table 1:** Inhibitory activity of LZ-101 against A549 xenograft tumor

Groups	Dose (mg/kg)	Inhibitory rate (%)
5-Fluorouracil	30	68.05**
LZ-101	10	40.86**
	20	55.62**

Each data point represents the mean SD of six mice

^**^P < 0.01 versus control group

**Table 2:** Hematology profile in non-tumor bearing athymic nude mice administered with normal saline. Two mice per group were used. Standard ranges were obtained in house from 100 normal BALB/c mice of 8–12 weeks age

Hematological parameters	Control	10 mg/kg	20 mg/kg	Standard
White blood cells (×10^3^ μl)	4.71/4.42	4.90/4.95	5.87/5.74	4.5–9.1
Red blood cells (×10^6^/μl)	9.76/10.70	10.51/9.84	10.25/9.30	7.51–16.1
Hemoglobin (g/dL)	14.5/13.0	15.2/14.2	14.9/13.9	12.8–16.1
Hematocrit (%)	44.8/41.8	47.9/45.3	46.9/44.7	34–50
Lymphocytes (%)	67.2/78.8	50.4/61.4	55.4/63.1	49–­82
Monocytes (%)	2.64/4.24	4.54/5.24	3.14/4.64	2–­8
Eosinophils (%)	0.24/0.34	0.04/0.74	0.54/0.64	0–­3
Basophils (%)	0.24/0.74	0.34/0.34	0.54/0.24	0–­3
Platelet (×10^3 ^μl)	507/410	560/483	526/744	115­–1037
Mean corpuscular volume (fL)	45.9/45.8	45.6/46.0	45.8/48.1	41–60
Mean corpuscular hemoglobin (pg)	14.9/14.3	14.5/14.4	14.5/14.9	13–19

## Discussion

LZ-101 is a derivative of danofloxacin, a fluoroquinolone antibiotic used in veterinary medicine. Previous studies indicated that high concentrations of danofloxacin displayed cytotoxic effects involved apoptosis and/or necrosis. Danofloxacin also induced a concentration-dependent increase in ROS production in renal tubular cells epithelial cell line (LLC-PK1)^23^. In the current study, we showed that LZ-101 had an effective anti-tumor activity in vitro and in vivo by stabilizing FOXO3a via blocking autophagy flux, eventually leading to mitochondrial-mediated apoptosis.

Primarily, we found that LZ-101 could induce apoptosis (Fig. 2a–c) and trigger caspase-9 activation and PARP cleavage in A549 cells. Moreover, LZ-101 increased the ratio of Bax/Bcl-2, the mitochondrial apoptotic indicator. These results suggested that LZ-101 induced apoptosis probably by activation of the mitochondrial apoptotic pathway. Therefore, we further investigated the effect of LZ-101 on mitochondrial apoptosis. The mitochondrial apoptotic pathway is comprised of a series of continuous processes. Our results showed that LZ-101 treatment remarkably decreased mitochondrial membrane potential, a marker for mitochondrial dysfunction in early apoptosis stage. During apoptosis, cytochrome *c* and AIF retained within mitochondria, are released from the intermembrane space into the cytosol. Afterwards, cytosolic cytochrome *c* binds to Apaf-1 to form the apoptosome which subsequently recruit procaspase-9 and facilitate its activation. Ultimately, caspase-9 efficiently cleaves and activates downstream caspase-3, leading to apoptosis^24^. AIF translocates from the mitochondria to the nucleus and causes chromatin condensation and DNA fragmentation^25^. Our results suggested that LZ-101 induced the release of cytochrome *c* and AIF from mitochondria into the cytosol, which was dependent on Bax expression (Fig. 2e, f). Taken together, our study implied that LZ-101 induced apoptosis via mitochondrial apoptotic pathway.

The Bcl-2 family proteins are most important regulators for apoptosis. This family consists of three subfamilies: anti-apoptotic members, pro-apoptotic members, and BH3-only members. Therein, the BH3-only members are normally triggered to mitochondrial translocation in response to apoptotic stimuli^26^. Once translocated into the mitochondria, they cause mitochondrial dysfunction and release pro-apoptotic proteins by interacting with other members of the Bcl-2 family^27^. Bim is a member of BH3-only proteins that bind to dynein light chain LC8 of microtubule complexes^28^. During apoptosis, Bim disassociates from the microtubule complex and translocates to the mitochondria, leading to the release of cytochrome *c*. When Bim gets into the mitochondria, the pro-apoptotic members of the Bcl-2 family, such as Bax and Bak, oligomerize into mitochondrial pores that are required to induce the release of pro-apoptotic proteins^29^. The members of the anti-apoptotic family, such as Bcl-2 and Bcl-xL, block the oligomerization of Bax and Bak but do not affect the translocation of the BH3-only proteins to the mitochondria^30–32^. In our study, LZ-101 promoted Bax and Bim expression and inhibited Bcl-2 expression (Figs. 2c and 3a). Transcriptional regulation of Bim may be important for apoptosis. It has been documented that Forkhead transcription factor FOXO3a, the most important transcription factor in FOXO family, induced Bim transcription^33^. Our study demonstrated that LZ-101 promoted FOXO3a expression, nuclear translocation and transcriptional activity. Further studies showed that LZ-101 inhibited FOXO3a degradation is independent of the proteasome pathway (Fig. 4a–c). Since the ubiquitin proteasome system and the autophagic lysosomal system are involved in the degradation of FOXO3a, we next studied the effect of LZ-101 on the autophagic lysosomal pathway. As shown in Fig. 4d, LZ-101 inhibited the co-localization of FOXO3a and LAMP-1. The combination of bafilomycin A1 and LZ-101 failed to further inhibit the degradation of FOXO3a (Fig. 4e). Further investigation of the effect of LZ-101 on autophagy revealed that LZ-101 could increase the accumulation of autophagosomes and block the autophagy flux (Fig. 5). Moreover, pharmacological or genetic inhibition of autophagosomes formation abolished FOXO3a stabilization and apoptosis induced by LZ-101 (Fig. 6). These results indicated that LZ-101 induced apoptosis by blocking autophagy-dependent FOXO3a degradation. Consistent with our in vitro studies, LZ-101 significantly suppressed the tumor growth of A549 inoculated xenograft mice with low toxicity in major organs. Mechanistically, we found that LZ-101 could induce apoptosis and regulate the expression of relevant proteins in tumor tissues (Fig. 7).

**Fig. 8:**
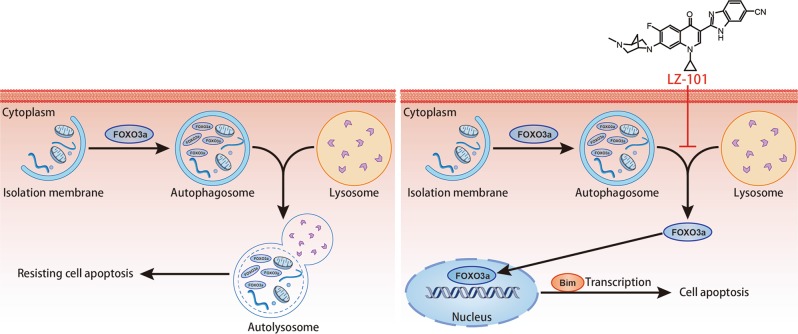
The possible mechanism of LZ-101 induced apoptosis in A549 cells. LZ-101 stabilized FOXO3a to induce mitochondrial apoptosis of A549 cells by inhibiting the degradation of autophagosomes containing-FOXO3a

In summary, LZ-101 displayed strong anti-tumor activity both in vitro and in vivo based on the mitochondrial apoptosis induced by up-regulation of Bim through stabilizing FOXO3a in autophagic lysosomal-dependent manner (Fig. 8). However, how FOXO3a escapes from autophagosomes to induce transcription remains unknown. Studies have shown that inhibition of autophagy in different means promoted the transcription of FOXO3a^16^. It was suggested that inhibition of autophagy induced FOXO3a activation was not a specific function of LZ-101. Therefore, the relationship between autophagy and FOXO3a activation needs further study and is underway in our laboratories. In conclusion, this study indicated that LZ-101 could be a potential effective agent with low toxicity for human non-small-cell lung carcinoma.

## Materials and methods

### Reagents

LZ-101 was dissolved in DMSO to make a 100-mM stock and stored at −20 °C until needed. The final concentration of DMSO did not exceed 0.1% throughout the study. 3-(4, 5-dimethylthiazol-2-yl)-2, 5-diphenyltetrazolium bromide (MTT), cycloheximide, 3-MA and diamidino-phenyl-indole (DAPI) were from Sigma (St. Louis, USA). Rapamycin was purchased form Cell Signaling Technology (Danvers, USA). Bovine serum albumin (BSA) was purchased from Roche (Mannheim, Germany).

### Antibodies

Antibodies to Bax, Bcl-2, caspase-9, Cyt *c*, LAMP-1, β-actin were from Santa Cruz Biotechnology (Dallas, USA). Antibodies to FOXO3a was from Bioworld (St Louis, USA). Antibodies to PARP, COX IV, AIF, Bim, LC3, ATG5, ATG7 were from Cell Signaling Technology (Danvers, USA) and antibody to SQSTM1/p62 were obtained from Abcam (Cambridge, UK). IRDye^TM^ 800 conjugated secondary antibodies were from Rockland Inc. (Philadelphia, USA) and diluted to the ratio of 1:15,000.

### Cell culture

Human non-small cell lung cancer cell line H460 and H1299 cells were cultured in RPMI-1640 medium (Gibco, Waltham, USA) and A549 cells were cultured in F-12 medium (Gibco, Waltham, USA) supplemented with 10% fetal bovine serum (Gibco, Waltham, USA), 100 U/ml penicillin and 100 U/ml streptomycin, cells were cultured in a humidified CO_2_ (5%) incubator (Thermo Forma, Waltham, USA) at 37 °C.

### MTT assay

Experiments were done in triplicate in a parallel manner for each concentration of LZ-101 used and the results are presented as mean ± SEM. Control cells were given culture media containing 0.1% DMSO. After incubation for 24 or 48 h, 20 μL of 5 mg/mL MTT was added to cells, and cells were incubated at 37 °C for another 4 h. The absorbance (A) was measured at 570 nm using an ELx800 automated microplate reader (BioTek Instruments, Inc.). The inhibitory ratio (%) was calculated using the following equation: inhibitory ratio = (1 – average absorbance of treated group/average absorbance of control group) × 100. IC_50_ was taken as the concentration that caused 50% inhibition of cell viability and was calculated by the Logit method.

### Annexin-V/PI staining

A549 cells were harvested, washed, and resuspended in PBS after LZ-101 treatment, then stained with the Annexin-V/PI Cell Apoptosis Detection Kit (KeyGen Biotech, Nanjing, China) according to the manufacturer’s instructions. Data acquisition and analysis were performed with a Becton Dickinson FACS Calibur flow cytometer using Cell-Quest software (BD Biosciences, Franklin Lakes, USA). The cells in early stages of apoptosis were Annexin-V positive and PI negative, whereas the cells in the late stages of apoptosis were both Annexin-V and PI positive.

### Mitochondrial transmembrane potential (*ΔΨ*_*m*_) assessment

The electrical potential difference across inner membrane (*ΔΨ*_*m*_) was monitored using the *ΔΨ*_*m*_-specific fluorescent probe JC-1 (Beyotime Institute of Biotechnology, Shanghai, China)^34^. The *ΔΨ*_m_-specific fluorescent probe JC-1 exists as a monomer with an emission at 530 nm (green fluorescence) at low membrane potential but forms J-aggregates with an emission at 590 nm (red fluorescence) at higher potentials. A549 cells were treated with LZ-101 for 24 h. Cells were harvested and incubated with JC-1 for 30 min at 37 °C in the dark, then resuspended in washing buffer and relative fluorescence intensities were monitored using flow cytometry (FACSCalibur, Becton Dickinson, USA) with settings of FL1 (green) at 530 nm and FL2 (red) 585 nm.

### Preparation of whole cell lysates and cytosolic and nuclear extracts

A549 cells were treated with LZ-101 at indicated concentrations for 24 h. The whole-cell lysates was prepared as described^35^. Nuclear and cytosolic protein extracts were prepared using a Nuclear/Cytosol Fractionation Kit (BioVision, Mountain View, CA) according to the manufacturer’s protocol. The cytosolic and nuclear fractions were reserved for immunoblot analysis. Final detection was performed with western blots.

### Mitochondrial fractionation

Mitochondrial fractionation kit (KeyGen Biotech, China) was used to get mitochondrial according to the following protocol. The cells were treated with different concentrations of LZ-101 for 24 h and 3.5 × 10^7^ cells were incubated with 1 mL ice-cold mitochondrial lyses buffer, then suspended and ground the cells with tight pestle on ice. The homogenate was subjected to centrifugation at 800 × *g* for 5 min at 4 °C to remove nuclei and unbroken cells, and then added 0.5 mL supernatant to the 0.5 mL Medium Buffer in the new 1.5 mL tube gently. After centrifugation at 15,000 × *g* for 10 min at 4 °C, the supernatant was carefully removed and collected as the cytosolic fraction and the remaining mitochondrial pellet was resuspended in the mitochondrial extraction buffer.

### Western blot analysis

The whole cell lysates, cytosolic extracts, nuclear extracts, and mitochondrial extracts were prepared as described above. Western blot analysis was carried out as described previously^35^. Protein samples were separated by 10% SDS-PAGE and transferred onto nitrocellulose membranes. The membranes were incubated with 1% BSA at 37 ℃ for 1 h and then indicated antibodies overnight at 4 ℃, followed by IRDye800 conjugated secondary antibody for 1 h at 37 ℃. Immunoreactive protein was detected with an Odyssey Scanning System (LI-COR Inc., Lincoln, Nebraska).

### Immunofluorescence microscopy

For confocal imaging of fixed cells, A549 cells were used. After the appropriate treatment, cells were fixed with 4% paraformaldehyde in PBS, permeabilized with 0.5% Triton X-100, and blocked with 3% BSA for 1 h. Samples was incubated with primary antibodies (diluted 1:100) overnight at 4 ℃. After washed, Alexa Fluor 488 donkey anti-rabbit IgG, Alexa Fluor 594 donkey anti-rabbit IgG were used as secondary antibodies (Invitrogen, CA, USA). Samples were observed and captured with a confocal laser scanning microscope (Olympus Corp., Tokyo, Japan).

### Cell transfection

GFP-LC3, mCherry-GFP-LC3 plasmid (Addgene, MA, USA), the siRNA targeting human FOXO3a, human ATG5, human ATG7 or control siRNA and the shRNA targeting human Bax or control shRNA with scrambled sequence were transfected using Lipofectamine 2000™ reagent (Invitrogen, CA, USA) according to the manufacturer’s instructions.

### Luciferase assay

A549 cells were seeded in 6-well plate, cultured for 24 h, and then transfected with 1 μg pGMFOXO-Lu (Genomeditech, Shanghai, China) and 0.05 μg pRL-TK Renilla (Beyotime, Nantong, China) with 10 μl Lipofectamine 2000 and incubated for 24 h at 37 ℃ with LZ-101. Cells were lysed with Promega passive lysis buffer and assayed by using Promega dual luciferase (Firefly luciferase/Renilla luciferase) kit. Luciferase intensity was detected with a Luminoskan Ascent (Thermo Fisher Scientific Inc. Finland).

### Anti-tumor effects in nude mice

Male BALB/c nude mice, 35–40 days old and with weight ranging from 18 to 22 g, were supplied by Shanghai Laboratory Animal Limited Company. The mice were maintained in a pathogen-free environment (23 ± 2 ℃ and 55 ± 5% humidity) on a 12 h light–12 h dark cycle with food and water supplied ad libitum throughout the experimental period. Mice were subcutaneously inoculated with 1 × 10^6^ A549 cells/nude mice. After 12–14 days, tumor sizes were determined using micrometer calipers, then nude mice with similar tumor volume (eliminate mice with tumors that are too large or too small) were randomly divided into four groups (6/group): one saline tumor control group; (i.v.) 5-fluorouracil 30 mg/ml/2 days group; (i.v.) LZ-101 10 mg/kg/2 days group; (i.v.) LZ-101 20 mg/ml/2 days group. At the end of 3 weeks, the mice were killed, and the tumor xenografts were removed and measured. Tumor volume (TV) was calculated using the following formula: TV (mm^3^) = *d*^2^ × *D*/2, where *d* and *D* are the shortest and the longest diameters, respectively. This study was approved in SPF Animal Laboratory of China Pharmaceutical University. Relative tumor volume (RTV) was calculated according to the equation: RTV = *V*_*t*_/*i*_0_, where *V*_0_ is the tumor volume at day 0 and *V*_*t*_ is the tumor volume at day *t*. And the evaluation index for inhibition was of relative tumor growth ratio T/C = TRTV/CRTV × 100%, where TRTV and CRTV represented RTV of treated and control groups, respectively.

### TUNEL assay

Apoptosis induction in the tissue specimen was analyzed by TUNEL assay. It was performed as per instructions given in situ cell death kit. The slides were photographed with a confocal laser scanning microscope (Fluoview FV1000, Olympus, Tokyo, Japan).

### Immunohistochemistry

The expression of FOXO3a, Bim, Bcl-2, Bax of the tissues of control and LZ-101 (20 mg/kg) treated groups was assessed by SP immunohistochemical method using a rabbit-anti-human monoclonal antibody and an Ultra-Sensitive SP kit (kit 9710 MAIXIN, Fuzhou, Fujian). Tissue sections (4 mm thick) were placed onto treated slides (Vectabond, Vector Laboratories, Burlingame, CA). Sections were heat fixed, deparaffinized and rehydrated through graded alcohols (100%, 95%, 85%, and 75%) to distilled water. Tissue sections were boiled in citrate buffer at high temperature for antigen retrieval, and treated with 3% hydrogen peroxide to block endogenous peroxidase activity. The slides were incubated with a protein-blocking agent (kit 9710 MAIXIN, Fuzhou, Fujian) prior to the application of the primary antibody, and then incubated with the primary antibody at 4 ℃ overnight. The tissues were then incubated with the secondary biotinylated anti-species antibody and labeled using a modification of the avidin–biotin complex immunoperoxidase staining procedure according to the UltraSensitive SP kit manual. Counterstaining was done with Harris hematoxylin. All reagents were supplied by MaixinBio Co. (Fuzhou, China).

### Statistical analysis

All data in different experimental groups were expressed as mean ± S.D. Data shown were obtained from at least three independent experiments. Statistical analysis was performed using an unpaired, two-tailed Student’s *t*-test. All comparisons were made relative to untreated controls and significance of difference is indicated as *p < 0.05 and **p < 0.01.
